# Proteome of seminal plasma and sperm associated with sperm survival following cryopreservation in the Red Wolf (*Canis rufus)*

**DOI:** 10.1038/s41598-025-21778-w

**Published:** 2025-10-29

**Authors:** Jasmin J. Celedon, Molly L. Corder, Meghann McConnell, Nucharin Songsasen, Jennifer B. Nagashima

**Affiliations:** Center for Species Survival, Smithsonian’s National Zoo & Conservation Biology Institute, 1500 Remount Rd., Front Royal, VA Washington, 22630 USA

**Keywords:** Seminal plasma, Sperm, Cryopreservation, Proteome, Wildlife conservation, Proteomics, Animal physiology, Reproductive biology

## Abstract

Understanding of the impacts of seminal plasma and sperm proteins on both animal and human fertility has expanded rapidly in the last decade. However, little is known about the reproductive proteomes of endangered species. In this study, we characterized for the first time the seminal plasma and sperm proteomes of the critically endangered Red Wolf and evaluated differences in proteomic signatures between ejaculates which experienced “High” versus “Baseline” (*i.e*. population average) sperm cryo-resilience. Highly cryo-resilient ejaculates (High_CR, *n* = 5) maintained > 50% sperm total motility relative to fresh samples following cryopreservation and post-thaw incubation, as well as acrosome integrity post-thaw. These samples had significantly higher expression of A1BG, APBB1, KRT1, KRT10, LOC609402 and LOC100685620 (AGP) proteins in seminal plasma, and significantly reduced expression of RHOA, NUP62, SMYD4, ARHGD1B, CAPG, CSTB, and CFL1 proteins in sperm compared with Baseline cryo-resilient samples (Base_CR, *n* = 5). Differences in protein expression indicated that alterations in physiological pathways including blood clotting cascades (seminal plasma) and both actin dynamics and immune pathways (sperm) are associated with sperm cryo-resilience. This new knowledge may prove useful in future optimization of semen cryopreservation and assisted reproductive technologies for the critically endangered Red Wolf.

**Supplementary Information:**

The online version contains supplementary material available at 10.1038/s41598-025-21778-w.

## Introduction

The American Red Wolf (*Canis rufus*) is a critically endangered canid native to the eastern portion of the United States, and a species of deep cultural significance to the Cherokee Nation and other Tribes^1^. There are an estimated 20 individuals in the wild (*in situ)* and ~280 managed individuals in the Association of Zoos and Aquariums (AZA) Saving Animals From Extinction (SAFE) population (*ex situ*)^2^. The *ex situ* population safeguards against the species’ extinction risks and provides a source of animal stock for reintroduction efforts into the wild; therefore, it is essential that every genetically valuable individual is represented in the population. While natural breeding is the gold standard for population propagation, it includes the risks of transporting individuals and potential for behavioral incompatibility. Assisted reproductive technologies (ARTs), including gamete cryopreservation and artificial insemination (AI), can eliminate these risks and have the potential to expand opportunities for genetic management of endangered species. AI using freshly collected semen has successfully produced offspring in the Red Wolf^3^, as well as other canid species including gray wolves (*Canis lupus)*^4^, and blue (*Vulpes lagopus)* and red foxes (*Vulpes vulpes)*^5,6^. Combined with sperm cryopreservation, AI has also successfully produced offspring in the critically endangered Mexican gray wolf^7^. Moreover, these ARTs can facilitate the movement of valuable genetics across both geographic distance and time, enabling the production of offspring beyond the natural lifespan of the sire.

Sperm cryobanking efforts in the Red Wolf began in the early 1990s and represent a substantial genetic reservoir for the species. There has also been significant research into the characterization of Red Wolf semen^8–10^ and optimization of cryopreservation protocols, including the influence of osmolarity and egg yolk concentration in semen extenders on survival and motility post-thaw^11^, assessment of various sperm cooling strategies prior to freezing^12,13^, and evaluation of culture media that support Red Wolf sperm capacitation and viability *in vitro*^14,15^. While significant progress has been made in advancing Red Wolf sperm cryopreservation, cryopreserved sperm motility can decrease by roughly 50% compared to fresh ejaculates; which challenges the use of such samples for AI^11,12^.

During development, sperm undergo chromatin remodeling which results in chromatin condensation, making nuclear DNA very compact and largely inaccessible to transcriptional machinery^16^. As a result, sperm are considered to be predominantly transcriptionally and translationally silent, which makes these cells largely dependent on proteins to shape cell function^17^. Thus, proteomic characterizations of sperm have greatly advanced our understanding of the molecular mechanisms underpinning sperm function^18^. The characterization of the protein composition of seminal plasma and sperm and their contributions to sperm function has rapidly expanded in domestic species such as the dog^19,20^, as well as in endangered mammals such as the black-footed ferret^21^ and Asian elephant^22^. Proteomic evaluations of seminal plasma from samples with high vs. low sperm freezability have identified candidate biomarkers (proteins) of cryo-resilience in ram^23^, carp^24^, and bull^25^ samples. These proteins have known roles in immune response, metabolism, and the inhibition of sperm capacitation^26^. Supplementation of seminal plasma or seminal plasma proteins have also been shown to support sperm function and cryo-resilience in some species^27,28^, though results have varied^29^. In the Asian elephant, supplementation with horse seminal plasma improved post-thaw sperm kinetics^22^. For sperm, a recent meta-analysis identified several oxidative stress-protective or antioxidant proteins, including glutathione-s-transferases (GSTs) and superoxide dismutase (SOD) linked across multiple species as associated with sperm cryo-tolerance^30^. Cryo-tolerant samples have also been associated with key sperm metabolism pathways^31^ and aquaporins, which are proposed to modulate cryo-tolerance via water and cryoprotective agent transport across sperm membranes^30^. This growing understanding is valuable to our ability to improve genetic banking efforts for both agriculturally relevant species and endangered wildlife. These findings demonstrate that large scale molecular characterizations of semen compartments could greatly improve cryopreservation success and subsequently assisted reproductive outcomes.

**Fig. 1:**
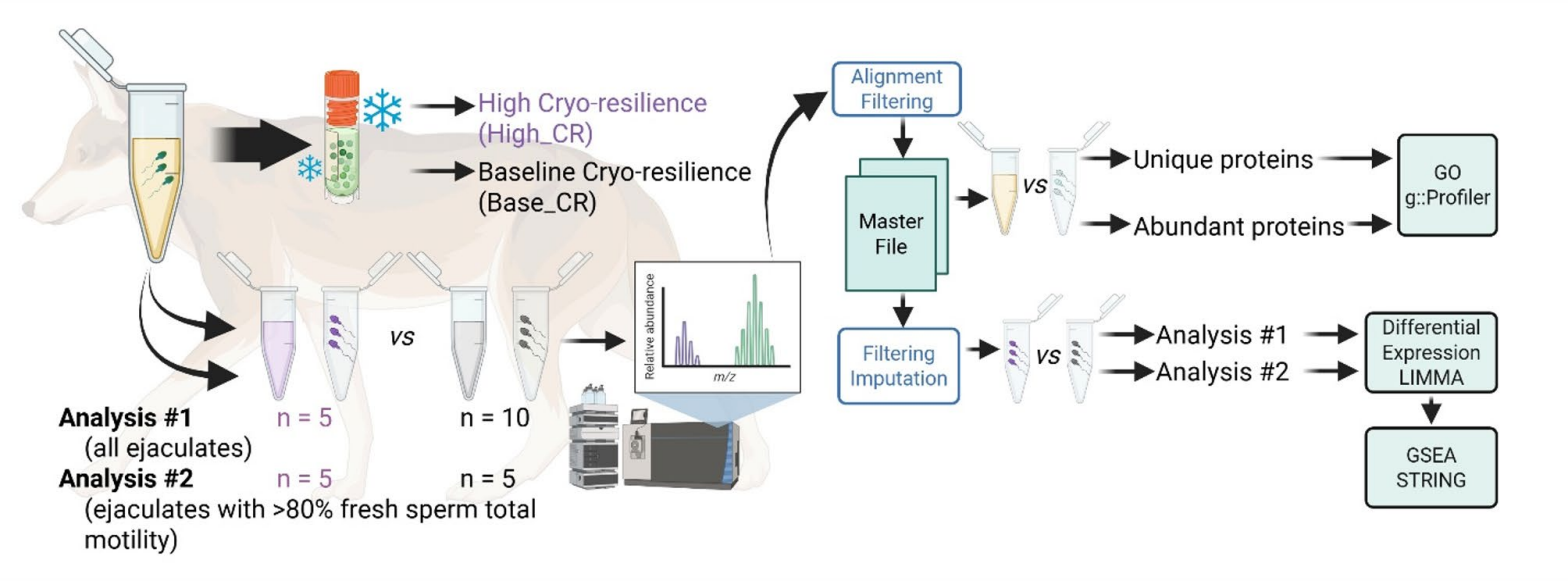
Study design schematic, created in BioRender.

In the present study, we characterize the proteome of fresh seminal plasma and sperm in Red Wolves and evaluate protein expression differences between highly cryo-resilient (High_CR) or baseline cryo-resilient (i.e. population average, Base_CR) ejaculates (Fig. 1). Through these analyses, we identified proteins which could be valuable supplements to optimize cryopreservation methods for this critically endangered species.

## Results

### Characterization of Red Wolf ejaculates at collection via electroejaculation

Semen samples were collected via electroejaculation from 15 adult male Red Wolves, with a single ejaculate collected per individual. All samples included in the study represented collections from animals with sufficient quantity and quality of ejaculate for cryopreservation. On average, wolves contributing to the study produced 5.78 ± 2.54 ml ejaculate with 3.18 ± 2.76 × 10^8^ total sperm and 81.67 ± 10.47% fresh total motility (Table 1**).**

**Table 1:** Red Wolf fresh semen collection characteristics.

RW ID	Age (y)	Right testis (cm)		Left testis (cm)		Volume (ml)	Sperm count	Fresh total motility
		L	W	L	W			
2079	8.73	3.19	2.36	3.70	2.05	2.5	9.20E + 07	90%
2107	6.84	-	-	-	-	6.0	3.14E + 08	90%
2151	6.79	3.28	2.41	3.69	2.10	4.2	5.05E + 08	90%
2163*	5.75	-	-	-	-	5.5	2.44E + 08	70%
2206*	4.79	3.50	3.02	3.75	3.15	11.5	3.70E + 08	65%
2229	3.80	3.30	2.70	3.40	2.80	6.5	1.14E + 09	90%
2230	3.80	3.60	3.10	3.50	2.90	4.3	3.57E + 08	90%
2240	3.82	2.92	2.45	3.10	2.16	4.5	3.10E + 08	80%
2241	3.82	3.56	2.31	3.56	2.67	10.0	5.80E + 08	90%
2269*	4.78	3.11	2.55	3.16	2.75	2.5	3.15E + 08	70%
2285	3.88	3.25	2.17	4.02	2.58	6.0	1.95E + 08	90%
2296	3.80	3.40	2.94	3.68	2.86	7.3	1.37E + 08	90%
2306	3.86	3.35	2.75	3.73	2.34	5.0	1.04E + 08	85%
2380*	1.87	2.95	2.02	3.00	1.90	7.5	1.23E + 08	70%
2381*	1.87	3.60	2.35	3.30	2.80	4.8	3.07E + 08	65%

* Indicating ejaculates with <80% fresh total sperm motility.

### Determination of high vs. baseline cryo-resilience (High_CR and Base_CR) via post-thaw Red Wolf sperm total motility, longevity, and membrane integrity

**Fig. 2:**
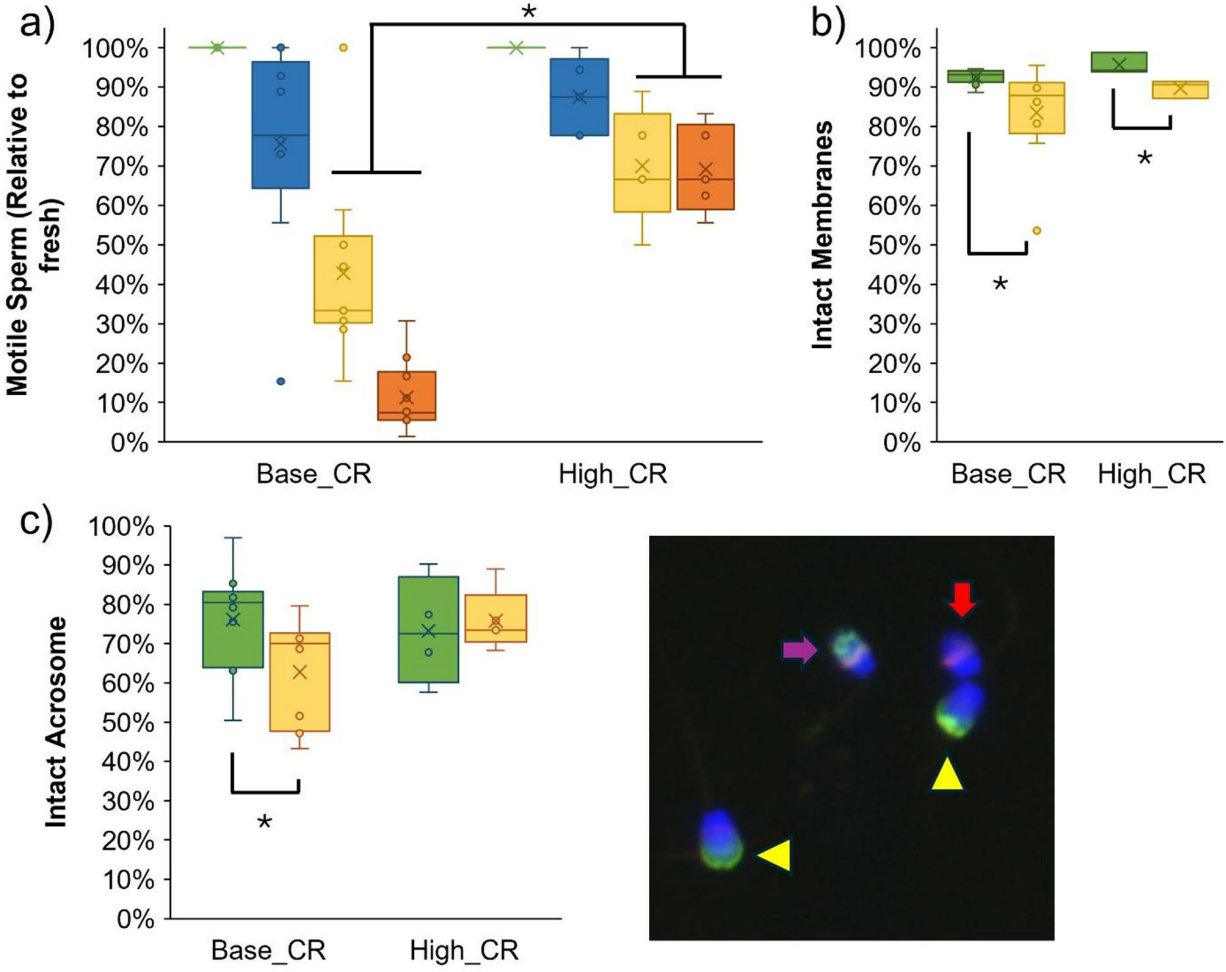
Survival and function of Red Wolf sperm following cryopreservation and thawing from all ejaculates (*n* = 15), with differing post-thaw sperm metrics between ejaculates with high (High_CR) versus baseline cryo-resilience (Base_CR), with (**a**) total motility and longevity relative to fresh sperm motility for each ejaculate; Fresh evaluations took place immediately following sperm collection, Chilled after at least 1 h of cooling at 4 °C in extender, Post-Thaw within 15 min after thawing, and 4 h Post-Thaw after 4 h incubation at 38 °C in mNCSU medium following thawing (**b**) percentage intact membranes via HOS test, and (**c**) acrosome status, with yellow arrowheads marking intact acrosomal membranes, purple arrow indicating a partial or damaged acrosome, and a red arrow indicating a sperm with absent acrosome. Asterisks (*) indicate statistically significant differences with *p* < 0.05.

Fresh total motility was analyzed in each ejaculate immediately following collection. Motility was also evaluated after sperm chilling (following centrifugation to remove seminal plasma, extension, cooling at 4 °C, and introduction of cryoprotectant, but prior to cryopreservation), post-thaw (after cryopreservation, storage, and within 15 min of thawing), and at 4 h post-thaw. As differences in fresh sperm total motility were observed between ejaculates, motility evaluations over time were normalized by each sample’s fresh total motility in Fig. 2a. Combining data from all ejaculates (*n* = 15), chilled sperm motility was not statistically different from fresh, but cryopreservation-thawing resulted in a significant reduction in total motility (*p* < 0.05), to 51.82 ± 24.28% motile sperm relative to fresh samples. This decrease was also observed following the 4 h post-thaw incubation, with 30.60 ± 29.76% total motile sperm relative to fresh samples. Differences were also observed in the percentage of sperm with intact membranes via hypo-osmotic swelling (HOS) test following cryopreservation and thawing across all evaluated ejaculates (*n* = 12), with 93.38 ± 2.45% intact in fresh and 85.09 ± 11.23% in post-thaw samples (*p* < 0.05). The percentage of sperm with intact acrosomal membranes post-thaw averaged 67.10 ± 13.13%; compared to 75.30 ± 13.04% in fresh samples from the same ejaculates (*p* > 0.05, *n* = 14).

A subset of the ejaculates (*n* = 5) displayed robust post-thaw longevity, maintaining > 50% total motility relative to their fresh motility even after 4 h incubation. These samples were considered to have high cryo-resilience (High_CR, Fig. 2a). Comparatively, the remaining ejaculates (*n* = 10) experienced a decline in sperm motility over incubation (“Baseline” samples, or Base_CR). Between High_CR and Base_CR groups, there were no differences in animal ages (avg 5.01 ± 1.65 and 4.32 ± 1.96 years, respectively), testes sizes (avg LxW (cm) = 3.35 × 2.55 and 3.44 × 2.54, respectively), nor fresh sperm plasma membrane integrity via HOS test (95.65 ± 2.71% and 92.63 ± 1.96%, respectively; Fig. 2b), or intact acrosome percentages (73.27 ± 13.92% and 76.11 ± 13.36% intact, respectively; Fig. 2c). Post-thaw, a reduction in intact plasma membranes was observed in both groups (*p* < 0.05); however, only the Base_CR ejaculates experienced a statistically significant reduction in percentage of intact acrosomal membranes following cryopreservation (*p* < 0.05). Nevertheless, it was noted that all ejaculates with < 80% fresh sperm total motility grouped with the Base_CR samples, representing slightly compromised function in these ejaculates even prior to cryopreservation. Therefore, two separate analyses were performed on the subsequent proteomic data. In the first (Analysis #1), all High_CR and Base_CR samples were included (*n* = 5 and 10 ejaculates, respectively), whereas in the second (Analysis #2), only samples which had ≥ 80% total motility at initial collection were included (animals without * in Table 1). Analysis #2 resulted in a High_CR group with 5 ejaculates (*n* = 5 animals, avg. age 5.01 ± 1.65 years, Fresh motility 88.0 ± 4.5%) and Base_CR with 5 ejaculates as well (*n* = 5 animals, avg. age 4.82 ± 2.19 years, Fresh motility 89.0 ± 2.2%), by excluding the ejaculates which had somewhat reduced sperm total motility at collection. No differences (*p* < 0.05) were observed in Fresh sperm intact plasma membranes or percentages of intact acrosomes between the two groups per Analysis #2.

## Identification of unique and highly abundant proteins in Red Wolf seminal plasma and sperm

**Fig. 3:**
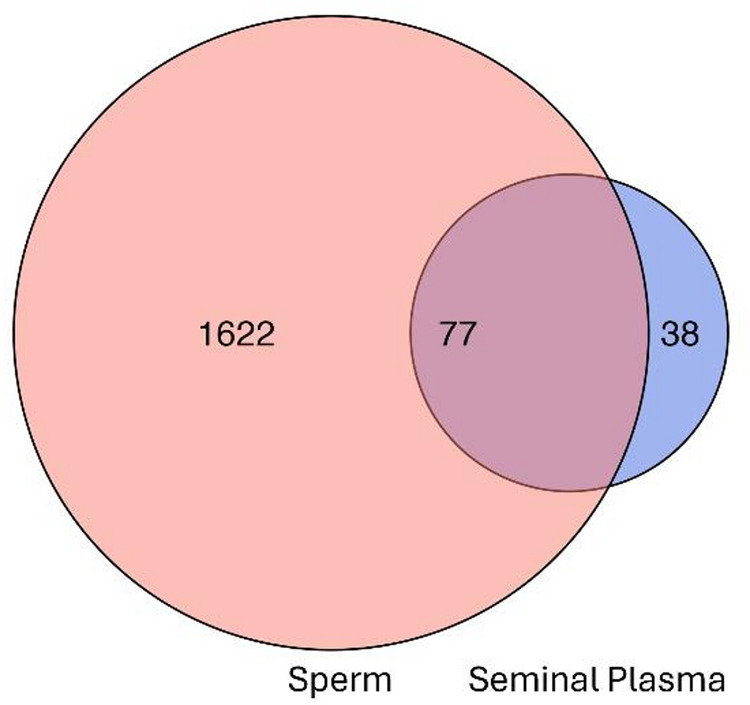
Comparison of unique and shared proteins between Red Wolf seminal plasma and sperm.

Following initial filtering, a total of 886 and 4517 proteins were identified in seminal plasma and sperm, respectively. Of these, 77 were found in both semen compartments, whereas 38 were unique to seminal plasma, and 1622 were unique to sperm (Fig. 3). The full list of unique and shared proteins from each semen compartment is in **Supplemental File 1**. Gene ontology analyses (GO) of unique seminal plasma and sperm proteins are provided in Supplemental File 5, Fig. S1 and S2, respectively. For additional validation of spectral results, a Western blot for cysteine-rich secretory protein 2 (CRISP2/LOC481834) was performed using sperm samples from a subset of the ejaculates (*n* = 6, with three from each cryo-resilience group). Similar patterns of expression were observed between mass spectrometry intensity results and immunoblotting protein expression (Supplemental File 5, Fig. **S3**). GO and KEGG analyses of the top 20 most abundant proteins in seminal plasma samples (listed in Table 2) identified enrichment in cholesterol metabolism and the lysosome. Highly abundant sperm proteins (Table 3) were involved in metabolic pathways, particularly glycolysis and carbon metabolism.

**Table 2:** Top 20 most abundant Red Wolf seminal plasma proteins.

	Gene ID	Name	Log2(Intensity)		
			Average	St dev	Median
1	*LTF*	Lactotransferrin	28.43	1.47	28.35
2	*ALB*	Albumin	28.36	1.06	28.05
3	*LOC119876012 | NPC2*	NPC intracellular cholesterol transporter 2	27.77	1.62	28.06
4	*KLNA1*	Kallikrein A1	27.70	0.93	28.25
5	*APOA1*	Apolipoprotein A1	26.72	1.56	26.59
6	*LCNL1*	Lipocalin like 1	26.67	1.69	26.84
7	*KLK2*	Kallikrein related peptidase 2	26.65	0.86	26.40
8	*LOC477072 | TF*	Serotransferrin	26.64	1.23	26.49
9	*LOC481834 | CRISP2*	Cysteine rich secretory protein 2	26.23	1.03	26.36
10	*DNASE1L3*	Deoxyribonuclease	26.19	0.82	26.27
11	*PIP*	Prolactin-induced protein	26.19	0.72	26.36
12	*CTSL*	Procathepsin L	26.19	0.42	26.11
13	*PTGDS*	Prostaglandin-H2 D-isomerase	26.07	1.56	26.48
14	*LRP2*	LDL receptor related protein 2	25.99	0.40	26.03
15	*CTSB*	Cathepsin B	25.80	0.44	25.87
16	*HEXB*	Beta-hexosaminidase	25.80	0.67	25.84
17	*KRT10*	Keratin, type I cytoskeletal 10	25.76	1.86	25.39
18	*PAM*	Peptidylglycine alpha-amidating monooxygenase	25.75	0.66	25.67
19	*LOC607874 | CRES*	Cystatin-C like	25.68	0.56	25.74
20	*PRSS1*	Peptidase S1 domain-containing protein | Serine protease 1	25.62	2.10	24.69

**Table 3:** Top 20 most abundant Red Wolf sperm proteins.

	Gene ID	Name	Log2 Intensity		
			Average	St Dev	Median
1	*AKAP4*	A-kinase anchoring protein 4	30.91	1.67	31.51
2	*LTF*	Lactotransferrin	30.79	2.25	30.92
3	*HK1*	Hexokinase 1	29.72	1.49	30.14
4	*KRT72*	Keratin 72	29.41	2.09	30.03
5	*AKAP3*	A-kinase anchoring protein 3	29.34	1.76	29.88
6	*GAPDHS*	Glyceraldehyde-3-phosphate dehydrogenase	29.09	1.95	29.76
7	*PTGES3*	ATP synthase subunit beta	29.09	1.55	29.31
8	*CCT8*	T-complex protein 1 subunit theta	29.08	1.41	29.39
9	*ACR*	Acrosin	29.07	1.78	29.58
10	*ALDOA*	Fructose-biphosphate aldolase	29.03	1.34	29.49
11	*SPAM1*	Hyaluronidase	28.97	1.70	29.46
12	*ATP5F1A*	ATP synthase subunit alpha	28.95	1.67	29.54
13	*MPO*	Myeloperoxidase	28.88	3.02	28.18
14	*KRT15*	Keratin 15	28.83	3.01	28.87
15	*NT5C1B*	5’-nucleotidase, cytosolid IB	28.68	1.53	29.07
16	*GK*	Glycerol kinase	28.62	1.67	29.14
17	*ACO2*	Aconitate hydratase, mitochondrial	28.62	1.78	29.09
18	*KRT75*	Keratin 75	28.60	2.09	28.27
19	*KRT10*	Keratin 10	28.59	1.59	28.91
20	*HSPA2*	Heat shock-related 70 kDa protein 2	28.55	1.59	29.00

## Differentially expressed seminal plasma and sperm proteins between High_CR and Base_CR Red Wolf ejaculates

Next, we performed bioinformatic analyses of seminal plasma and sperm proteomes, comparing High_CR and Base_CR samples. In **Analysis #1** (including all ejaculates), differential expression analyses of both seminal plasma and sperm (Supplemental File 6), yielded 5 and 8 proteins reaching statistical significance, respectively. For seminal plasma, these included keratin 1 (KRT1), keratin 10 (KRT10), alpha-1-acid glycoprotein 1 (LOC100685620/AGP), and granulin (GRN), which were upregulated in High_CR samples, and Mannosyl-oligosaccharide 1,2-alpha-mannosidase IA (MAN1A1) which had reduced expression in High_CR samples compared with Base_CR samples. For sperm, all eight differentially expressed proteins had reduced expression in High_CR compared with Base_CR. Differentially expressed proteins included Nucleoporin p62 (NUP62), SET and MYND domain-containing protein 4 (SMYD4), Napsin A aspartic peptidase (NAPSA), Rho GDP-dissociation inhibitor beta (ARHGDIB), Heteroous nuclear ribonucleoprotein A3 (LOC119881680/HNRNPA3), Globin domain-containing protein (HBQ1), Basic leucine zipper and W2 domain-containing protein 1 (BZW1), and Capping actin protein, gelsolin like (CAPG) (*p* < 0.05).

**Fig. 4:**
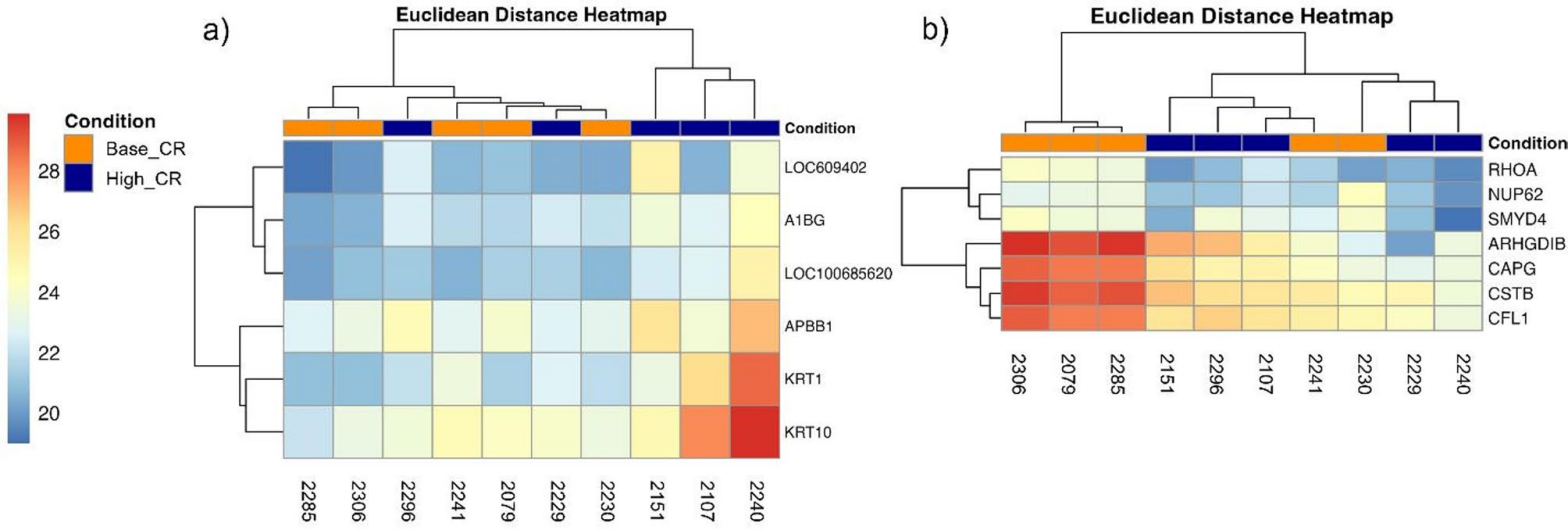
Heatmaps of differentially expressed proteins in Red Wolf** (a) **seminal plasma and **(b) **sperm, from High_CR (*n* = 5, with blue overlay) versus Base_CR (*n* = 5, with orange overlay) groups, utilizing protein expression data from ejaculates with ≥ 80% total motility in fresh sperm.

Limiting our bioinformatic evaluations to ejaculates with fresh total motility ≥ 80% (**Analysis #2**), seminal plasma evaluations identified KRT1, KRT10, AGP as well as alpha-1-B glycoprotein (A1BG), hemoglobin subunit beta-like (LOC609402), and Amyloid beta A4 precursor protein-binding family B member 1 (APBB1) with higher expression in High_CR (*p* < 0.05, Heatmap Fig. 4a). For sperm, seven proteins with reduced expression in High_CR versus Base_CR samples were identified, including SMYD4, NUP62, ARHGDIB, and CAPG, as well as Cystatin-B (CSTB), Ras Homolog Family Member A (RHOA) and Cofilin 1 (CFL1) (Heatmap Fig. 4b, *p* < 0.05).

### STRING pathway analyses of Red Wolf seminal plasma and sperm protein expression in High_CR versus Base_CR ejaculates

**Fig. 5:**
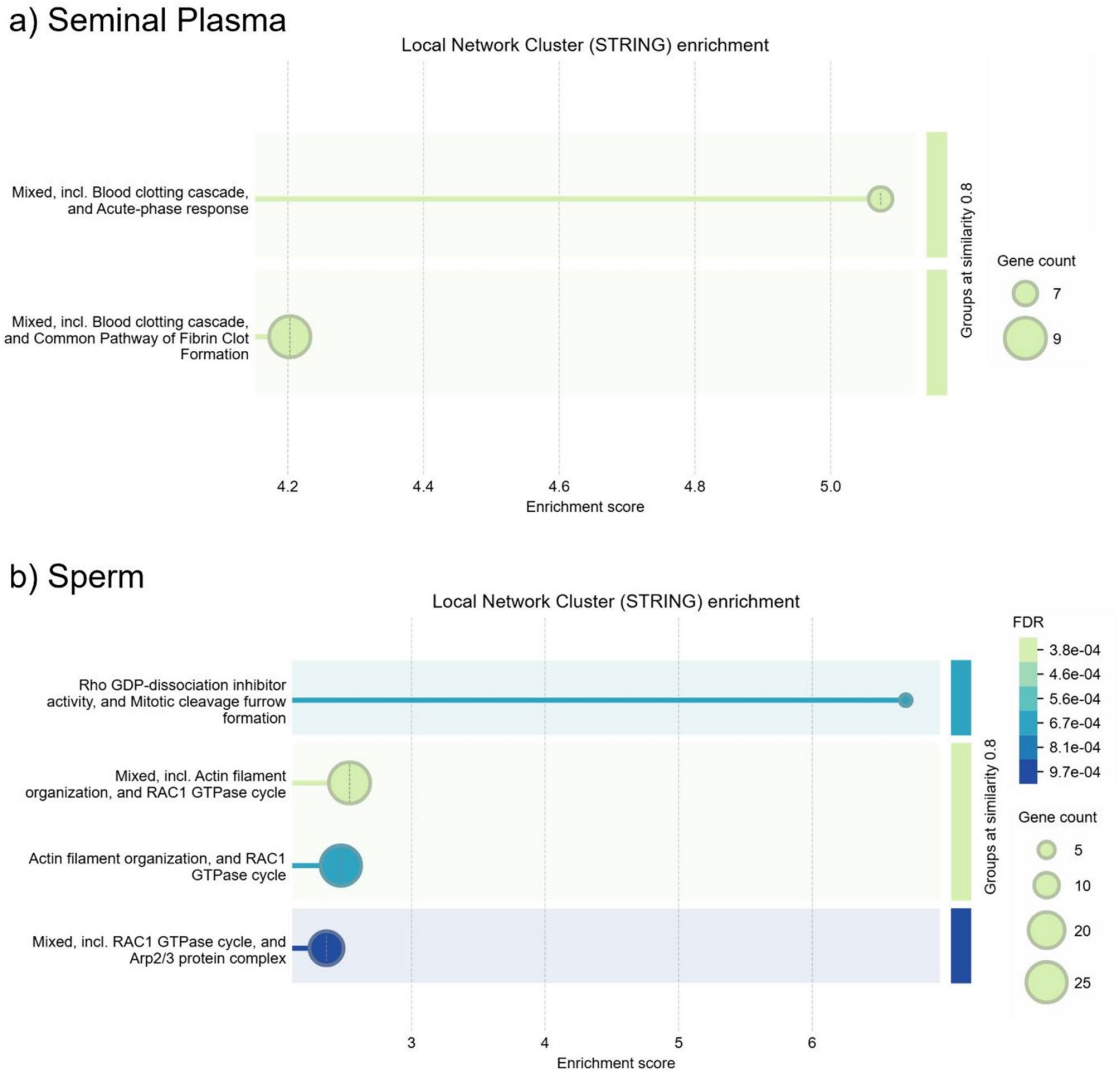
STRING value-ranked functional enrichment results based on logFC for Red Wolf** (a)** seminal plasma, and** (b) **sperm from High_CR (*n* = 5) versus Base_CR (*n* = 5) groups, utilizing protein expression data from ejaculates with ≥ 80% total motility in fresh sperm.

Seminal plasma and sperm protein expressions in High_CR and Base_CR ejaculates were next evaluated for functional enrichment. A value-ranked list was generated based on log fold changes between the groups then STRING pathway analyses performed. In seminal plasma, 109 of 115 proteins were mapped to the domestic dog proteome (https://version-12-0.string-db.org/organism/9615). STRING enrichments included Mixed pathways, including blood clotting cascade, acute-phase response, and common pathway of fibrin clot formation (CL:17822 and 17820; Fig. 5a). These pathways were highly represented at the top of the value-ranked input, indicating that they were enriched in the High_CR seminal plasma compared with Base_CR samples. In sperm, 1575 of 1699 proteins were mapped to the dog proteome, identifying enrichments in Rho GDP pathways and actin filament organization (CL:21991, 21899, 21904, and 21906), shown in Fig. 5b. STRING value-ranked analysis of sperm proteins also identified a high number of hits for proteins associated with the Neutrophil degranulation Reactome pathway (CFA-6798695), which were clustered toward the bottom of the value-ranked input, or with reduced expression in the High_CR sperm compared with Base_CR samples.

## Discussion

Understanding of the impacts of seminal plasma and sperm proteins on both animal and human fertility is growing. However, little is known about the reproductive proteomes of some of our most endangered species. Here, we characterize for the first time the seminal plasma and sperm proteomes of the critically endangered Red Wolf, toward the goal of improving our understanding of the physiology of the species and the development of improved assisted reproductive technologies. The current study identified several proteins in both seminal plasma and sperm which were differentially expressed between ejaculates experiencing “High” versus “Baseline” cryo-resilience, based on post-thaw total motility and acrosome integrity. The differences in protein expression in both ejaculate compartments indicated alterations in actin dynamics and immune response pathways influencing cryo-resilience in the Red Wolf.

Of the proteins which were present in 100% of ejaculates, we identified 77 proteins shared between seminal plasma and sperm sample types, with 38 and 16228 unique proteins to each type, respectively. Several of the most highly abundant proteins, such as lactotransferrin, were expected due to their described abundance in the seminal plasma of other species including the domestic dog^20,32^. In the Asian elephant, LTF has been shown to be differentially expressed in fresh ejaculates from males with ‘high’ versus ‘low’ motility sperm^33^. While we observed high expression of LTF in both seminal plasma and sperm in the present study, it did not differ in expression between the two cryo-resilience groupings for either semen compartment. That said, the handling process involving both the removal of seminal plasma and potential changes to sperm proteins during cryopreservation/thawing may reduce LTF levels in post-thaw Red Wolf sperm, as has been demonstrated in the ram^34^. In the bull, stallion, and ram, LTF supplementation during sperm cryopreservation has had beneficial effects ranging from improved survival and membrane integrity^35–37^. Future evaluation of how the various steps of the cryopreservation process impact proteins like LTF, with key associations with sperm function in other species, may assist in improving the cryopreservation process moving forward.

In this study, multiple key proteins were differentially expressed between High_CR and Base_CR groups in seminal plasma or sperm. Focusing on the results of Analysis #2, the differentially expressed seminal plasma proteins (A1BG, AGP, KRT1, KRT10, APBB1, and LOC609402) all had significantly elevated expression in High_CR samples compared with Base_CR, and several have described roles in fertility. For example, seminal plasma alpha-1-B glycoprotein (A1BG) has also been linked to differences in sperm cryo-tolerance in rams^38^, and has been shown to modify the function of cysteine rich secretory protein (CRISP2)^39^. Notably, CRISP2 was a highly abundant protein in Red Wolf sperm in the current study - which was biologically validated by Western blotting in our sperm samples – and is a key regulator of sperm motility and the ability to undergo an acrosome reaction in the mouse^40^. Though CRISP2 expression did not differ between High_CR and Base_CR sperm in our study, the interaction between seminal plasma A1BG expression and sperm CRISP2 function in Red Wolves warrants future exploration. Alpha-1-acid glycoprotein (AGP) has also been suggested as a potential marker for human male infertility^41^. AGP is an acute-phase protein with multiple functions in inflammation, including modulating the activities of neutrophils^42,43^. AGP produced by the bovine oviduct has been shown to suppress neutrophil phagocytosis of sperm, an effect which was eliminated if the AGP was desialynated^44^. In the current study, AGP was elevated in seminal plasma from High_CR ejaculates compared with Base_CR; although, no evaluations of specific glycosylation/sialynation abundances were assessed. This may indicate a protective role for the abundant AGP on Red Wolf sperm and underscores the need for future research to assess post-translational modifications among seminal proteins. In our pathway analyses, however, we observed enrichment in Mixed pathways, including blood clotting cascade, acute-phase response, and common pathway of fibrin clot formation, which likely reflects the similarities others have noted between serum and seminal plasma molecular composition^45,46^.

In contrast with the seminal plasma, all seven of the proteins differentially expressed in sperm (SMYD4, NUP62, ARHGDIB, CAPG, CSTB, RHOA and CFL1) had significantly reduced expression in High_CR samples. Many of these proteins also have previously been described in sperm proteomic research in other species. For example, elevated expression of CSTB, a cysteine protease inhibitor, has been observed in boars with poor resistance to cooling to 5 °C compared with high cooling-resilience^47^, matching the pattern we observed in our cryo-resilient Red Wolf sperm in present study. Moreover, several of the differentially expressed proteins had well-established roles in actin dynamics, which was reflected in the STRING pathway analysis. Modulation of actin polymerization underlies many of the changes that must occur for sperm capacitation^48^. In particular, the reorganization of actin into its more polymerized, filamentous structure (F-actin) compared with monomeric, globular actin (G-actin) is needed for acrosomal exocytosis. This shift is promoted by additional proteins identified in the current study, including CFL1, which inactivates actin depolymerization activities when phosphorylated, RHOA^49^, which modulates CFL1 phosphorylation, and ARHGD1B, known to inactivate Rho proteins by inhibiting GDP/GTP exchange^50^. Finally, CAPG, an F actin capping protein^51^ is also a modulator of actin dynamics. Together, the reduced expression in each of these proteins in the High_CR ejaculates indicate significant alterations in actin dynamics, likely resulting in higher monomeric compared with filamentous actin in the highly cryo-resilient Red Wolf sperm. Intriguingly, addition of recombinant RhoA protein to rooster semen during cryopreservation has been demonstrated to increase sperm motility and acrosome integrity following cryopreservation and warming^52^, which was proposed to be due to F-actin stabilization of the sperm head cytosol^53^. This suggests modulation of actin polymerization dynamics via the RhoA/C pathway is an interesting candidate for improving Red Wolf sperm cryopreservation success in the future.

In the sperm, STRING Reactome analysis identified ‘neutrophil degranulation’ as a pathway enriched in Base_CR samples compared with the High_CR samples. Neutrophil degranulation, along with phagocytosis and NETosis (neutrophil extracellular traps/NETs)^54^, are specific neutrophil responses that modulate the immune-mediated host defense. Neutrophils have been demonstrated to trap sperm, produce ROS that damage sperm, and/or ultimately facilitate their removal from the female reproductive tract^55^, with seminal plasma contributions supporting sperm escape from NETs in some species^56,57^. In the domestic dog, polymorphonuclear neutrophils and NETs have been found to be abundant in ejaculates from healthy animals, and NETs have been shown to trap dog sperm and negatively impact viability and acrosome integrity^58^. Recently, Dash et al. suggested that the reduced sperm motility observed in clinically healthy men following recovery from COVID-19 may have been due to continued inflammation/neutrophil activation resulting in increased ROS in the reproductive tract^59^. The results of the current study suggest that the neutrophil signals endogenous to Red Wolf semen are associated with the sperm’s reduced motility and acrosome integrity following cryopreservation-thawing. However, it is not clear if neutrophils are contributing to the poor sperm resilience directly via degranulation, or if their presence is indicative of an existing immune challenge which is indirectly affecting sperm function. Considering the beneficial impact of seminal plasma observed in other species and the enriched expression of neutrophil-modulating AGP in seminal plasma from the highly cryo-resilient ejaculates, exploration of neutrophil activity in Red Wolf semen and potential remediation strategies represent a priority for understanding the interaction between immune function in the male reproductive tract and sperm function/cryo-resilience.

In sum, the study characterizes for the first time the seminal plasma and sperm proteomes of the American Red Wolf. Several proteins/pathways differed between ejaculates which experienced high versus baseline cryo-resilience, as evaluated by post-thaw sperm total motility, longevity, and membrane integrity. Seminal plasma from highly cryo-resilient ejaculates were enriched in coagulation cascade pathways, proteins associated with CRISP-family protein function, and immune response modulating AGP. Sperm from these ejaculates also had reduced expression of RhoA/C pathway member proteins, whereas neutrophil degranulation Reactome pathway enrichment was identified in sperm with baseline cryo-resilience. Further evaluation of these differentially expressed proteins will improve our understanding of their roles in supporting sperm function and cryo-resilience; and support genetic banking and management efforts for the critically endangered Red Wolf.

## Methods

### Animals

Ejaculates were collected from 15 adult male Red Wolves (single collection per animal) from various SAFE institutions during the 2022 and 2023 breeding seasons (February-March). Individuals were selected for collection based on health status and genetic priority for banking (based on mean kinship value). Animals were exposed to natural photoperiod and fed diets consisting of commercially available dry kibble and/or whole carcass, with water provided ad libitum. All procedures were done in accordance with USFWS guidelines (permit #ES 61239D-1), AZA SAFE program recommendations, the Smithsonian Institution Animal Care and Use Committee approval (#SI-21025), and ARRIVE guidelines.

## Semen collection and processing

Red Wolves were fasted 24 h before semen collection. On collection day, animals were captured and transported a short distance from their outdoor enclosure to an indoor collection site via a crate. Anesthesia drugs varied by institutional veterinary staff but commonly consisted of 0.4 mg/kg butorphanol and 0.04 mg/kg medetomidine. Before collection, testicular dimensions (length, width and firmness) were evaluated – with the exception of two individuals who were not measured due to collection procedure time-constraints. The penis was subsequently exteriorized, washed with sterile saline and dabbed dry sterile gauze. The bladder was flushed using a 5 Fr, 55.8 cm red rubber catheter (Coviden/Kendall) and warmed saline to minimize urine contamination. Semen was collected using electroejaculation using a 1.9 cm probe and between 60 and 90 stimulations of 2–6 V over two series, with a 5–7-minute rest period between. The intensity of stimulation was adjusted based on the individual’s physical response, as appropriate. Ejaculates were collected in sterile polypropylene cups and fractions pooled by individual, unless there was urine contamination or a major difference in sperm concentration or motility among ejaculate fractions. Ejaculate volumes ranged from 2.5 to 11.5 ml per individual (Table 1). A small volume (~ 30 ul) of pooled semen was used to assess sperm concentration, motility (percentage of motile sperm out of total, evaluated via phase contrast microscope over at least 5 areas), and pH, or fixed in 4% paraformaldehyde for morphology/acrosome integrity evaluations. Moreover, where possible, an additional 20 µl of raw ejaculate was utilized for hypo-osmotic swelling test (HOS) test, described below. Only ejaculates which met cryopreservation criteria (≥ 60% motility, ≥ 50 × 10^6^ total sperm, ≥ 2.5 ml ejaculate volume) were included in the proteomic study. Animals were allowed to recover from anesthesia under the care of veterinary staff.

For proteomic evaluations, a 400–500 µl aliquot of the ejaculate was also removed and separated into seminal plasma and spermatozoa. Briefly, the ejaculate was centrifuged at 2,000 g for 10 min to pellet the sperm. Seminal plasma supernatant was subsequently filtered through a 0.22 μm syringe filter to remove any cell contamination, then immediately frozen on dry ice and stored at −80 °C until proteomic analysis. The sperm pellet was washed via resuspension in phosphate buffered saline (PBS), and centrifugation again at 2,000 g for 10 min, then the pellet was also frozen on dry ice and stored at −80 °C until proteomic evaluations.

### Sperm cryopreservation, warming, and incubation

For the remaining ejaculate, the semen was then centrifuged at 300 g for 5 min to remove the seminal plasma. Sperm samples were resuspended in TRIS-egg yolk extender at a concentration of 200 × 10^6^ sperm/ml and cooled in a refrigerator for at least 2 h (to ~ 5 °C). Cooled extender with 8% glycerol was subsequently added to the chilled samples in a dropwise manner over a period of 15 min, for a final concentration of 100 × 10^6^ sperm/ml in 4% glycerol extender. The extended sperm was then pipetted using precooled pipette tips into depressions in dry ice, forming pellets of cryopreserved sperm. After 3 min, the pellets were plunged into liquid nitrogen and loaded into labeled cryovials for storage in liquid nitrogen.

Sperm pellets (2 pellets per ejaculate) were warmed by transfer directly from liquid nitrogen into 1 mL of warmed mNCSU^14^ per pellet. The supernatant containing cryoprotectant was removed, and the cryopreserved-warmed sperm pellet resuspended in warmed mNCSU to a concentration of 7.5 × 10^6^ sperm/ml, as determined via hemocytometer. Following warming, washing, and re-suspension, a small aliquot of each sample was fixed with 4% paraformaldehyde for acrosome analyses. Additionally, 20 µl was utilized for HOS test and the remainder incubated at 5% CO_2_, 5% O_2_ and 90% N_2_ at 37 °C in a humidified chamber on an EVOS Fl Auto 2 microscope (ThermoFisher Scientific, USA) in a welled plate. At 0, 2, and 4 h post-warming, videos were recorded of each sample with each video being roughly 20 s and at 4 different areas of the well (~ 400 sperm per area). Videos were evaluated for total motility by a single observer.

## Hypo-osmotic swelling test (HOST)

Sperm were diluted 1:10 in HOS test solution (25 mM sodium citrate dihydrate, 75 mM fructose) and incubated at 37 °C for 30–60 min before evaluation^60^. Sperm with straight tails with no evidence of swelling were considered ‘damaged’, whereas those with kinked or coiled tails, or enlarged midpieces indicating functional membranes in response to hypo-osmotic conditions were considered ‘intact’. At least 200 sperm were evaluated per animal and time point (*n* = 12 ejaculates) and reported as percentage of membrane-intact sperm.

### Acrosome integrity evaluation

Red Wolf sperm acrosomes were evaluated via fluorescent microscopy (*n* = 14 ejaculates). Briefly, fixed sperm slides were washed with PBS, permeabilized with 0.1% Triton X in PBS for 15 min, then incubation in 10 µg/ml PNA and 5 µg/ml Hoechst 33,343 (ThermoFisher Scientific) for 1 h at room temperature before mounting with Prolong anti-fade mountant (ThermoFisher) and glass coverslip. At least 200 sperm per animal and time point were evaluated as ‘Intact’, or having a smooth, continuous acrosomal surface, ‘damaged’, with pigmented or irregular acrosomal membranes, or ‘absent’.

### Label-free nano liquid chromatography tandem mass spectrometry (LC-MS/MS)

Sample processing and mass spectrometry was performed by Creative Proteomics (https://www.creative-proteomics.com). Briefly, 1 µg of each processed sample was loaded and analyzed with Ultimate 3000 nano UHPLC system (ThermoFisher Scientific, USA). The flow rate was 250 nL/min. The mobile phase A used 0.1% formic acid in water and phase B used 0.1% formic acid in 80% acetonitrile. The mass spectrometry was performed between 300 and 1,650 m/z at the resolution 60,000 at 200 m/z.

### Alignment and filtering

Mass spectrometry.raw files were aligned to the domestic dog (*Canis lupus familiaris*) proteome (Uniprot UP000805418, https://www.uniprot.org/proteomes/UP000805418) in MetaMorpheus^61^, using default settings. Briefly, the calibration settings included: 15 mm precursor mass tolerance, 25 mm product mass tolerance, trypsin protease, 2 maximum mixed cleavages, and common fixed and variable modifications. Search settings included 5 ppm precursor mass tolerance and 20 ppm product mass tolerance, with 2 maximum modifications per peptide, a minimum peptide length of 7, and 0.01 Q-value threshold. Quantification utilized the FlashLFQ approach. The quantified peptide and protein groups were subsequently filtered in RStudio (v. 4.4.2, R Core Team, 2024). A false discovery rate (FDR) of 5% was applied to all quantified peptides. The most abundantly expressed proteins were identified as those identified in all samples of each given semen compartment, based on average log2 intensity signal/expression (Tables 2 and 3). For subsequent analyses, the data were further filtered to remove proteins that had more than 30% missing values. Data were normalized via log2 transformation and imputation performed using missForest algorithm^62^ to minimize bias during the subsequent differential expression analyses.

### Gene ontology and differential expression analysis

Cryo-resilience treatment groups were designated primarily based on post-thaw sperm motility results, where a natural separation between groups was observed after 4 h incubation (> 50% versus ≤ 30% relative motility). The unique proteins present from each semen compartment were used for Gene Ontology (GO) analysis in g: Profiler using the domestic dog (*Canis lupus familiaris)* as background and standard analyses (Only annotated genes, g:SCS threshold) (Supplemental File 5, Figs. S1 and S2 for seminal plasma and sperm, respectively). The R package Linear models of microarray data (LIMMA)^63,64^ was used for differential expression to compare the seminal plasma and fresh spermatozoa data between the “High” and “Baseline” cryo-resilience groups (Data and scripts in Supplemental Files S2-4). Heatmaps were constructed using the R package dendextend^65^ and Euclidian distances.

### Gene set enrichment analysis

Functional enrichment of gene networks identified by differentially expressed proteins were analyzed via the String database (v12.0)^66^ (https://string-db.org/) in seminal plasma and sperm, and were subsequently ranked via fold-change, allowing for 5% FDR.

### Western blotting

To validate spectral findings, Western blotting was performed to determine the presence of sperm CRISP2 (LOC481834) in a subset of ejaculates. This protein was selected as it was identified in every sperm sample in the mass spectrometry results in high abundance, and has previously been described in domestic dog epididymal sperm^67^. Briefly, sperm were lysed via repeated freeze-thaw in liquid nitrogen, and protein concentration evaluated using Qubit protein assay. Then, 8 µg of Red Wolf sperm protein (*n* = 6 animals from the 15 included in the study) were loaded into a 4%−20% Mini Protean TGX Stain- Free Gel (BioRad Laboratories, Hercules, CA, USA) with standard Precision Plus Protein™ Dual Color Standards (1610394; BioRad Laboratories, Hercules, CA, USA). Positive control sample included epididymal sperm obtained from domestic dog testes from routine castration at a local spay/neuter clinics. Proteins were transferred to nitrocellulose membranes with the Trans-Blot Turbo transfer system (BioRad Laboratories, Hercules, CA, USA) and transfer success and loaded protein validation evaluated via Ponceau staining^68^. Immunoblotting was performed with 1 h blocking with 5% nonfat milk in TBST, overnight incubation at 4 °C with CRISP2 Polyclonal antibody (PA5-109590; 1:1000; ThermoFisher Scientific, Waltham, MA, USA), and 3 h incubation at room temperature with Goat Anti-Rabbit IgG (H + L) HRP Conjugate secondary antibody (1706515; 1:2500; BioRad Laboratories, Hercules, CA, USA). Imaging was done using SuperSignal™ West Pico PLUS Chemiluminescent Substrate (34580; ThermoFisher Scientific, Waltham, MA, USA) with the G: Box chemi XRQ (Sygene). Protein expression was quantified via pixel intensity evaluation of imaged blots, normalized to Ponceau intensity, in ImageJ (v1.54 g)^69^.

### Statistical analyses

All data were reported as mean ± standard deviation. Differences between High_CR and Base_CR ejaculates’ animal characteristics (ages, testis dimensions) and sperm metrics (total motility, percentages of intact plasma and acrosomal membranes) were evaluated via nonparametric Kruskal Wallis test in R Studio^70^. The R package Linear models of microarray data (LIMMA)^63,64^ was used for differential expression to compare the seminal plasma and fresh sperm data between the High_CR and Base_CR groups, where p values < 0.05 were considered to have reached statistical significance.

## Supplementary Information

Below is the link to the electronic supplementary material.

Supplementary Material 1

Supplementary Material 2

Supplementary Material 4

Supplementary Material 6

Supplementary Material 3

Supplementary Material 5

## Data Availability

Data are available in the manuscript and supplementary information files.Supplemental File 1: Unique proteins present in Red Wolf seminal plasma and spermSupplemental File 2: Imputed dataframe for seminal plasma “RW_Masterfile_SPlasma_Impdf”Supplemental File 3: Imputed dataframe for sperm “RW_Masterfile_SpzFresh_Impdf”Supplemental File 4: R scripts for LIMMA analyses, “1_DifferentialExpression_SeminalPlasma” and “1_Differential Expression_SpzFresh”Supplemental File 5: Supplemental figures, including GO analyses of unique proteins to each semen compartment, CRISP2 validation, and STRING Reactome pathway enrichment of Red Wolf sperm proteins using p-value ranked list.Supplemental File 6: LIMMA outputs for both Analyses 1 and 2 for both semen compartments.
