# Supplementary material for: Proteome of seminal plasma and sperm associated with sperm survival following cryopreservation in the Red Wolf (Canis rufus)
Source: Sci Rep. 2025 Oct 29;15:37882. doi: 10.1038/s41598-025-21778-w (PMC12572227; doi:10.1038/s41598-025-21778-w)
Supplement: Supplementary file 4 — Supplementary Material 6 [file 41598_2025_21778_MOESM4_ESM.docx]

---

title: "1_DifferentialExpression_Seminal Plasma"

author: "Molly Corder"

date: "`r Sys.Date()`"

output:

html_document:

toc: yes

toc_depth: 3

number_sections: true

toc_float:

collapsed: false

smooth_scroll: true

fig_caption: yes

---

# Introduction

In this script, we will run a differential expression analysis using the

**LIMMA** package.

Differential expression analysis allows us to answer the question:

"Are there differences in the proteomic profiles between groups A & B?"

## Read in data

Load required libraries

```{r}

library(tidyverse)

library(ggplot2)

library(limma)

```

Load data

# Plasma

```{r}

data <- readr::read_tsv(file = "RW_Masterfile_SPlasma_Impdf.tsv")

meta <- read.csv(file = "ExperimentalDesign_RWSemen.csv" )

```

## Filter

1. Remove columns that we don't need

2. Check for duplicate Gene IDs (rows)

Remove any data that is not GeneID or intensity columns

```{r}

#Remove columns 1 and 3

data <- data[, -c(1,3)]

```

Now let's inspect the "Gene" column in the "data" data frame.

Notice | separating out multiple (and sometimes overlapping Gene IDs).

We're going to keep only the first gene ID and remove any others.

```{r}

data$Gene <- gsub("\\|.*", "", data$Gene)

```

These rows are now missing gene IDs.

3, 24-26, 36, 44, 51, 55, 79, 82, 106, 112, 126, 128

```{r}

#Remove rows missing Gene IDs

data <- data %>%

filter(Gene != "NA")

data <- data %>%

filter(Gene != "")

# Check for duplicates in Gene column

duplicated(data$Gene)

#None are true

length(unique(data$Gene))

```

Save data to be transposed and re-read into R for PCA.

```{r}

readr::write_csv(data, "RW_SPlasma_forPCA_AllAnimals.csv")

#removing individuals below the 80% initial motility threshold (Analysis #2)

data2 <-data[, -c(5,6,11,15,16)]

readr::write_csv(data2, "RW_SPlasma_forPCA_SubsetAnimals.csv")

```

```{r}

meta <- meta[,-c(3:5)] #Drop unnecessary columns

#removing individuals below the 80% initial motility threshold (Analysis #2)

meta2 <- meta[-c(4,5,10,14,15),]

```

# Differential Expression Analysis

Let's run LIMMA!

Linear Models for Microarray Data

Limma workflow involves:

1. Importation of expression data

2. Design matrix

Construct a design matrix that encodes the experimental design to specify

how each sample is related to different experimental conditions.

3. Linear Model Fitting:

Fit a linear model which uses gene-wise linear models (lmFit)

4. Empirical Bayes Moderation

Apply empirical Bayes moderation which shrinks the standard errors and

improves detection of differentially expressed compounds

5. Generate topTable

Provides ranked list of genes and related statistical metrics/p-values

```{r}

#Start with Full Dataset analysis

#Design matrix that encodes experimental design

design <- model.matrix(~0+factor(meta$Condition), data = meta)

colnames(design) <- c("Control", "High")

#Fit a linear model using gene-wise linear models

fit <- lmFit(data, design)

cont.matrix <- makeContrasts(Condition = Control - High, levels=design)

fit2 <- contrasts.fit(fit, cont.matrix)

#Use empirical Bayes moderation to shrink standard errors and improve detection

#of differentially expressed compounds

fit2 <- eBayes(fit2)

#Generate topTable with ranked genes and statistical metrics

tpCondition <- topTable(fit2,number=dim(data)[1])

write_tsv(tpCondition, "tpCondition_SPlasma_AllAnimals.tsv")

write_csv(tpCondition, "tpCondition_SPlasma_AllAnimals.csv")

```

```{r}

#repeat for Subset Data (only including animals with over 80% initial motility, aka Analysis #2)

#Design matrix that encodes experimental design

design <- model.matrix(~0+factor(meta2$Condition), data = meta2)

colnames(design) <- c("Control", "High")

#Fit a linear model using gene-wise linear models

fit_A2 <- lmFit(data2, design)

cont.matrix <- makeContrasts(Condition = Control - High, levels=design)

fit2_A2 <- contrasts.fit(fit_A2, cont.matrix)

#Use empirical Bayes moderation to shrink standard errors and improve detection

#of differentially expressed compounds

fit2_A2 <- eBayes(fit2_A2)

#Generate topTable with ranked genes and statistical metrics

tpCondition2 <- topTable(fit2_A2,number=dim(data2)[1])

write_tsv(tpCondition2, "tpCondition_SPlasma_SubsetAnimals.tsv")

write_csv(tpCondition2, "tpCondition_SPlasma_SubsetAnimals.csv")

```

Now open your tpCondition.tsv file and review the following:

***topTable Outputs***

**Gene**: Gene ID encoding for experimentally detected protein

**logFC**: log fold change value - shows direction of protein expression

between cases and controls

**AveExpr**: overall expression of gene levels.

**t**: t-statistic used to assess differential expression.

Higher absolute values indicate stronger evidence.

**P.Value**: p-value for differential expression

NOT adjusted for multiple testing

**adj.P.Val**: p-value for differential expression adjusted for multiple testing

by default adjustment method = Benjamini-Horchberg

**B**: log odds ratio *Bayesian measure) of differential expression probability.

# Volcano Plot, Subset Animals

```{r}

set.seed(1)

tpCondition2 <- read.csv("tpCondition_SPlasma_SubsetAnimals.csv")

```

Set filtering parameters. Filter out FC values between 0.5 and 1.5.

Below log2(0.5) = downregulated.

Above log(1.5) = upregulated.

Therefore, other values are considered NOT differetially expressed.

```{r}

#Annotate according to differential expression (prepare background gene sets)

tpCondition2 <- tpCondition2 %>%

mutate(diffexpressed = case_when(

logFC > 0.58 & P.Value < 0.05 ~ 'UP',

logFC < -1 & P.Value < 0.05 ~ 'DOWN',

P.Value > 0.05 ~ 'NO'

))

#RE_dfClass <- tpClassRE %>%

# mutate(diffexpressed = case_when(

# logFC > 0 & adj.P.Val < 0.05 ~ 'UP',

# logFC < 0 & adj.P.Val < 0.05 ~ 'DOWN',

# adj.P.Val > 0.05 ~ 'NO'

# ))

#Drop NA values

tpCondition2 <- tpCondition2 %>%

filter(diffexpressed != 'NA')

#Count number of up and downregulated genes

tpCondition2 %>%

count(diffexpressed)

#When including fold change values and regular p-value, there are no compounds

#to visualize with differential expression data (high vs low).

```

# Heatmaps

Load required Libraries

```{r setup 1, include=FALSE}

library(tidyverse)

library(ggplot2)

library(factoextra)

library(corrplot)

library(limma)

library(ggrepel)

library(RColorBrewer)

library(pheatmap)

library(dendextend)

```

```{r}

set.seed(222)

```

```{r}

#Focus on the subset data

meta2 <- meta2[order(match(meta2$FileName,

colnames(data2)[2:11])),]

```

Load metadata

```{r}

rownames(meta2) <- meta2$FileName;

meta2$FileName <- NULL

newmeta <- as.data.frame(meta2)

all.equal(rownames(newmeta), colnames(data2)[-1])

```

Significant p-value from tpCondition:

A1BG

LOC100685620

LOC609402

KRT1

APBB1

KRT10

```{r}

data2 <- data2 %>%

dplyr::mutate(Gene = sub("\\|.*", "", Gene))

```

Note: these were upregulated in the control group (aka down-reg in High by 2 FC)

```{r}

#Filter out significant proteins (by p-value)

DE <- data2 %>%

filter(Gene %in% c("A1BG", "LOC100685620", "LOC609402", "KRT1", "APBB1", "KRT10"))

```

Make a Dendrogram

```{r}

DE.labels <- DE$Gene

DE_data = DE[2:11]

DE_data_std = scale(DE_data)

DE_dist = dist(DE_data_std)

#HC clustering algorithm

hc.out_DE <- hclust(DE_dist, method = "complete")

hc.out_DE

plot(hc.out_DE)

#Clusters

DE.clusters <- cutree(hc.out_DE, k = 2)

#Visualize

rownames(DE_data_std) <- paste(DE$Gene, 1:dim(DE)[1], sep = "")

fviz_cluster(list(data = DE_data_std, cluster = DE.clusters)) +

theme_minimal()

```

```{r}

DE <- as.data.frame(DE)

gene_names <- DE$Gene

gene_data <- DE[, -1]

DE <- as.data.frame(lapply(DE[-1], as.integer))

```

```{r}

# Calculate distances with different methods

euclidean_dist_rows <- dist(gene_data, method = "euclidean")

manhattan_dist_rows <- dist(gene_data, method = "manhattan")

correlation_dist_rows <- as.dist(1 - cor(gene_data, method = "pearson"))

# Perform hierarchical clustering for rows

complete_clusters_euclidean_rows <- hclust(euclidean_dist_rows, method = "complete")

complete_clusters_manhattan_rows <- hclust(manhattan_dist_rows, method = "complete")

complete_clusters_correlation_rows <- hclust(correlation_dist_rows, method = "complete")

# Calculate distances for columns

euclidean_dist_cols <- dist(t(gene_data), method = "euclidean")

manhattan_dist_cols <- dist(t(gene_data), method = "manhattan")

correlation_dist_cols <- as.dist(1 - cor(t(gene_data), method = "pearson"))

# Perform hierarchical clustering for columns

complete_clusters_euclidean_cols <- hclust(euclidean_dist_cols, method = "complete")

complete_clusters_manhattan_cols <- hclust(manhattan_dist_cols, method = "complete")

complete_clusters_correlation_cols <- hclust(correlation_dist_cols, method = "complete")

#Manhattan Distance Heatmap

pheatmap(as.matrix(gene_data),

cluster_rows = complete_clusters_manhattan_rows,

cluster_cols = complete_clusters_manhattan_cols,

main = "Manhattan Distance Heatmap",

show_rownames = TRUE,

labels_row = gene_names,

fontsize_col = 4,

fontsize = 4)

#Euclidean Distance Heatmap

DEGs <- pheatmap(as.matrix(gene_data),

cluster_rows = complete_clusters_euclidean_rows,

cluster_cols = complete_clusters_euclidean_cols,

show_rownames = TRUE,

labels_row = gene_names,

main = "Euclidean Distance Heatmap",

fontsize_col = 4,

fontsize = 4)

DEGs

ggsave("DiffExpresHeatmap_v1_SPlasma.png", DEGs)

#Same plot, but overlay CCP class metadata

annotation_col <- data.frame(Condition = newmeta$Condition)

row.names(annotation_col) <- colnames(gene_data)

class_colors <- c(Control = "darkorange", High = "blue4")

annotation_colors <- list(Condition = class_colors)

# Generate the heatmap with annotations

DEGswCondition <- pheatmap(as.matrix(gene_data),

cluster_rows = complete_clusters_euclidean_rows,

cluster_cols = complete_clusters_euclidean_cols,

annotation_col = annotation_col,

annotation_colors = annotation_colors,

show_rownames = TRUE,

labels_row = gene_names,

main = "Euclidean Distance Heatmap",

fontsize_col = 9,

fontsize = 9)

DEGswCondition

ggsave("DEGswCondition_Heatmap_SPlasma.png", DEGswCondition)

```

---

title: "1_DifferentialExpression_SpzFresh"

author: "Molly Corder"

date: "`r Sys.Date()`"

output:

html_document:

toc: yes

toc_depth: 3

number_sections: true

toc_float:

collapsed: false

smooth_scroll: true

fig_caption: yes

---

# Introduction

In this script, we will run a differential expression analysis using the

**LIMMA** package.

Differential expression analysis allows us to answer the question:

"Are there differences in the proteomic profiles between groups A & B?"

## Read in data

Load required libraries

```{r}

library(tidyverse)

library(ggplot2)

library(limma)

```

Load data

# Plasma

```{r}

data <- readr::read_tsv(file = "RW_Masterfile_SpzFresh_Impdf.tsv")

meta <- read.csv(file = "ExperimentalDesign_RWSemen.csv")

```

## Filter

1. Remove columns that we don't need

2. Check for duplicate Gene IDs (rows)

Remove any data that is not GeneID or intensity columns

```{r}

#Remove columns 1 and 3

data <- data[, -c(1,3)]

```

Now let's inspect the "Gene" column in the "data" data frame.

Notice | separating out multiple (and sometimes overlapping Gene IDs).

We're going to keep only the first gene ID and remove any others.

```{r}

data$Gene <- gsub("\\|.*", "", data$Gene)

```

These rows are now missing gene IDs.

168, 187, 191, 206, 250-2, 293, 435, 462, 563, 584, 606, 675, 680, 732, 769, 780, 911, 922, 976, 984, 1076, 1085, 1087, 1123, 1133, 1158-9, 1180, 1199, 1251-3, 1262, 1271, 1286, 1304, 1310, 1350, 1364, 1374, 1402, 1405, 1440, 1502, 1514, 1544, 1645, 1667, 1680, 1684, 1724, 1770, and 1775

```{r}

#Remove rows missing Gene IDs

data <- data %>%

filter(Gene != "NA")

data <- data %>%

filter(Gene != "")

# Check for duplicates in Gene column

duplicated(data$Gene)

#There are several true!

length(unique(data$Gene))

```

One gene is duplicated

```{r}

duplicates <- duplicated(data$Gene)

print(duplicates)

#Find rows where duplicate is TRUE

duplicate_rows <- data[duplicates, ]

duplicate_rows

#Now go to data and search for: KRT75

#Notice it appears three times

```

## Handle duplicate Gene IDs

We have 26 instances of duplicate Gene IDs. You could use this

to aggregate on the median:

```{r}

data <- data %>% group_by(Gene) %>%

summarise_if(is.numeric, function(x) median(x, na.rm = T))

```

Save data to be transposed and re-read into R for PCA.

```{r}

readr::write_tsv(data, "RW_SpzFresh_forPCA_AllAnimals.tsv")

#removing individuals below the 80% initial motility threshold (Analysis #2)

data2<- data[,-c(5,6,11,15,16)]

readr::write_csv(data2, "RW_SpzFresh_forPCA_SubsetAnimals.csv")

```

```{r}

meta <- meta[,-c(3:5)] #Drop unnecessary columns

#removing individuals below the 80% initial motility threshold (Analysis #2)

meta2 <- meta[-c(4,5,10,14,15),]

```

# Differential Expression Analysis

Let's run LIMMA!

Linear Models for Microarray Data

Limma workflow involves:

1. Importation of expression data

2. Design matrix

Construct a design matrix that encodes the experimental design to specify

how each sample is related to different experimental conditions.

3. Linear Model Fitting:

Fit a linear model which uses gene-wise linear models (lmFit)

4. Empirical Bayes Moderation

Apply empirical Bayes moderation which shrinks the standard errors and

improves detection of differentially expressed compounds

5. Generate topTable

Provides ranked list of genes and related statistical metrics/p-values

```{r}

#Design matrix that encodes experimental design

design <- model.matrix(~0+factor(meta$Condition), data = meta)

colnames(design) <- c("Control", "High")

#Fit a linear model using gene-wise linear models

fit <- lmFit(data, design)

cont.matrix <- makeContrasts(Condition = Control - High, levels=design)

fit2 <- contrasts.fit(fit, cont.matrix)

#Use empirical Bayes moderation to shrink standard errors and improve detection

#of differentially expressed compounds

fit2 <- eBayes(fit2)

#Generate topTable with ranked genes and statistical metrics

tpCondition <- topTable(fit2,number=dim(data)[1])

write_tsv(tpCondition, "tpCondition_SpzFresh_AllAnimals.tsv")

write_csv(tpCondition, "tpCondition_SpzFresh_AllAnimals.csv")

```

Repeat for Analysis #2 (including only animals with >80% initial motility)

```{r}

#Design matrix that encodes experimental design

design <- model.matrix(~0+factor(meta2$Condition), data = meta2)

colnames(design) <- c("Control", "High")

#Fit a linear model using gene-wise linear models

fit2 <- lmFit(data2, design)

cont.matrix <- makeContrasts(Condition = Control - High, levels=design)

fit2_A2 <- contrasts.fit(fit2, cont.matrix)

#Use empirical Bayes moderation to shrink standard errors and improve detection

#of differentially expressed compounds

fit2_A2 <- eBayes(fit2_A2)

#Generate topTable with ranked genes and statistical metrics

tpCondition2 <- topTable(fit2_A2,number=dim(data2)[1])

write_tsv(tpCondition2, "tpCondition_SpzFresh_SubsetAnimals.tsv")

write_csv(tpCondition2, "tpCondition_SpzFresh_SubsetAnimals.csv")

```

Now open your tpCondition.tsv file and review the following:

***topTable Outputs***

**Gene**: Gene ID encoding for experimentally detected protein

**logFC**: log fold change value - shows direction of protein expression

between cases and controls

**AveExpr**: overall expression of gene levels.

**t**: t-statistic used to assess differential expression.

Higher absolute values indicate stronger evidence.

**P.Value**: p-value for differential expression

NOT adjusted for multiple testing

**adj.P.Val**: p-value for differential expression adjusted for multiple testing

by default adjustment method = Benjamini-Horchberg

**B**: log odds ratio *Bayesian measure) of differential expression probability.

# Volcano Plot

```{r}

set.seed(1)

tpCondition2 <- read.csv("tpCondition_SpzFresh_SubsetAnimals.csv")

```

Set filtering parameters. Filter out FC values between 0.5 and 1.5.

Below log2(0.5) = downregulated.

Above log(1.5) = upregulated.

Therefore, other values are considered NOT differetially expressed.

```{r}

#Annotate according to differential expression (prepare background gene sets)

tpCondition2 <- tpCondition2 %>%

mutate(diffexpressed = case_when(

logFC > 0.58 & P.Value < 0.05 ~ 'UP',

logFC < -1 & P.Value < 0.05 ~ 'DOWN',

P.Value > 0.05 ~ 'NO'

))

#RE_dfClass <- tpClassRE %>%

# mutate(diffexpressed = case_when(

# logFC > 0 & adj.P.Val < 0.05 ~ 'UP',

# logFC < 0 & adj.P.Val < 0.05 ~ 'DOWN',

# adj.P.Val > 0.05 ~ 'NO'

# ))

#Drop NA values

tpCondition2 <- tpCondition2 %>%

filter(diffexpressed != 'NA')

#Count number of up and downregulated genes

tpCondition2 %>%

count(diffexpressed)

#When including fold change values and regular p-value, there are no compounds

#to visualize with differential expression data (high vs low).

```

Load required Libraries

```{r setup 1, include=FALSE}

library(tidyverse)

library(ggplot2)

library(factoextra)

library(corrplot)

library(limma)

library(ggrepel)

library(RColorBrewer)

library(pheatmap)

library(dendextend)

```

```{r}

set.seed(222)

```

```{r}

#Focus on the subset data

meta2 <- meta2[order(match(meta2$FileName,

colnames(data2)[2:11])),]

```

Load metadata

```{r}

rownames(meta2) <- meta2$FileName;

meta2$FileName <- NULL

newmeta <- as.data.frame(meta2)

all.equal(rownames(newmeta), colnames(data2)[-1])

```

Significant p-value from tpCondition:

SMYD4

NUP62

ARHGDIB

CAPG

CSTB

RHOA

CFL1

```{r}

data2 <- data2 %>%

dplyr::mutate(Gene = sub("\\|.*", "", Gene))

```

Note: these were upregulated in the control group (aka down-reg in High by 2 FC)

```{r}

#Filter out significant proteins (by p-value)

DE <- data2 %>%

filter(Gene %in% c("SMYD4", "NUP62", "ARHGDIB", "CAPG", "CSTB", "RHOA", "CFL1"))

```

Make a Dendrogram

```{r}

DE.labels <- DE$Gene

DE_data = DE[2:11]

DE_data_std = scale(DE_data)

DE_dist = dist(DE_data_std)

#HC clustering algorithm

hc.out_DE <- hclust(DE_dist, method = "complete")

hc.out_DE

plot(hc.out_DE)

#Clusters

DE.clusters <- cutree(hc.out_DE, k = 2)

#Visualize

rownames(DE_data_std) <- paste(DE$Gene, 1:dim(DE)[1], sep = "")

fviz_cluster(list(data = DE_data_std, cluster = DE.clusters)) +

theme_minimal()

```

```{r}

DE <- as.data.frame(DE)

gene_names <- DE$Gene

gene_data <- DE[, -1]

DE <- as.data.frame(lapply(DE[-1], as.integer))

```

```{r}

# Calculate distances with different methods

euclidean_dist_rows <- dist(gene_data, method = "euclidean")

manhattan_dist_rows <- dist(gene_data, method = "manhattan")

correlation_dist_rows <- as.dist(1 - cor(gene_data, method = "pearson"))

# Perform hierarchical clustering for rows

complete_clusters_euclidean_rows <- hclust(euclidean_dist_rows, method = "complete")

complete_clusters_manhattan_rows <- hclust(manhattan_dist_rows, method = "complete")

complete_clusters_correlation_rows <- hclust(correlation_dist_rows, method = "complete")

# Calculate distances for columns

euclidean_dist_cols <- dist(t(gene_data), method = "euclidean")

manhattan_dist_cols <- dist(t(gene_data), method = "manhattan")

correlation_dist_cols <- as.dist(1 - cor(t(gene_data), method = "pearson"))

# Perform hierarchical clustering for columns

complete_clusters_euclidean_cols <- hclust(euclidean_dist_cols, method = "complete")

complete_clusters_manhattan_cols <- hclust(manhattan_dist_cols, method = "complete")

complete_clusters_correlation_cols <- hclust(correlation_dist_cols, method = "complete")

#Manhattan Distance Heatmap

pheatmap(as.matrix(gene_data),

cluster_rows = complete_clusters_manhattan_rows,

cluster_cols = complete_clusters_manhattan_cols,

main = "Manhattan Distance Heatmap",

show_rownames = TRUE,

labels_row = gene_names,

fontsize_col = 4,

fontsize = 4)

#Euclidean Distance Heatmap

DEGs <- pheatmap(as.matrix(gene_data),

cluster_rows = complete_clusters_euclidean_rows,

cluster_cols = complete_clusters_euclidean_cols,

show_rownames = TRUE,

labels_row = gene_names,

main = "Euclidean Distance Heatmap",

fontsize_col = 4,

fontsize = 4)

DEGs

ggsave("DiffExpresHeatmap_v1_Sperm.png", DEGs)

#Same plot, but overlay CCP class metadata

annotation_col <- data.frame(Condition = newmeta$Condition)

row.names(annotation_col) <- colnames(gene_data)

class_colors <- c(Control = "darkorange", High = "blue4")

annotation_colors <- list(Condition = class_colors)

# Generate the heatmap with annotations

DEGswCondition <- pheatmap(as.matrix(gene_data),

cluster_rows = complete_clusters_euclidean_rows,

cluster_cols = complete_clusters_euclidean_cols,

annotation_col = annotation_col,

annotation_colors = annotation_colors,

show_rownames = TRUE,

labels_row = gene_names,

main = "Euclidean Distance Heatmap",

fontsize_col = 9,

fontsize = 9)

DEGswCondition

ggsave("DEGswCondition_Heatmap_Sperm.png", DEGswCondition)

```

```
