# Supplementary material for: Proteome of seminal plasma and sperm associated with sperm survival following cryopreservation in the Red Wolf (Canis rufus)
Source: Sci Rep. 2025 Oct 29;15:37882. doi: 10.1038/s41598-025-21778-w (PMC12572227; doi:10.1038/s41598-025-21778-w)
Supplement: Supplementary file 5 — Supplementary Material 3 [file 41598_2025_21778_MOESM5_ESM.docx]

Supplemental File 3

**Supplemental Figure S1. GO analysis of proteins unique to Red Wolf seminal plasma from g:Profiler**.


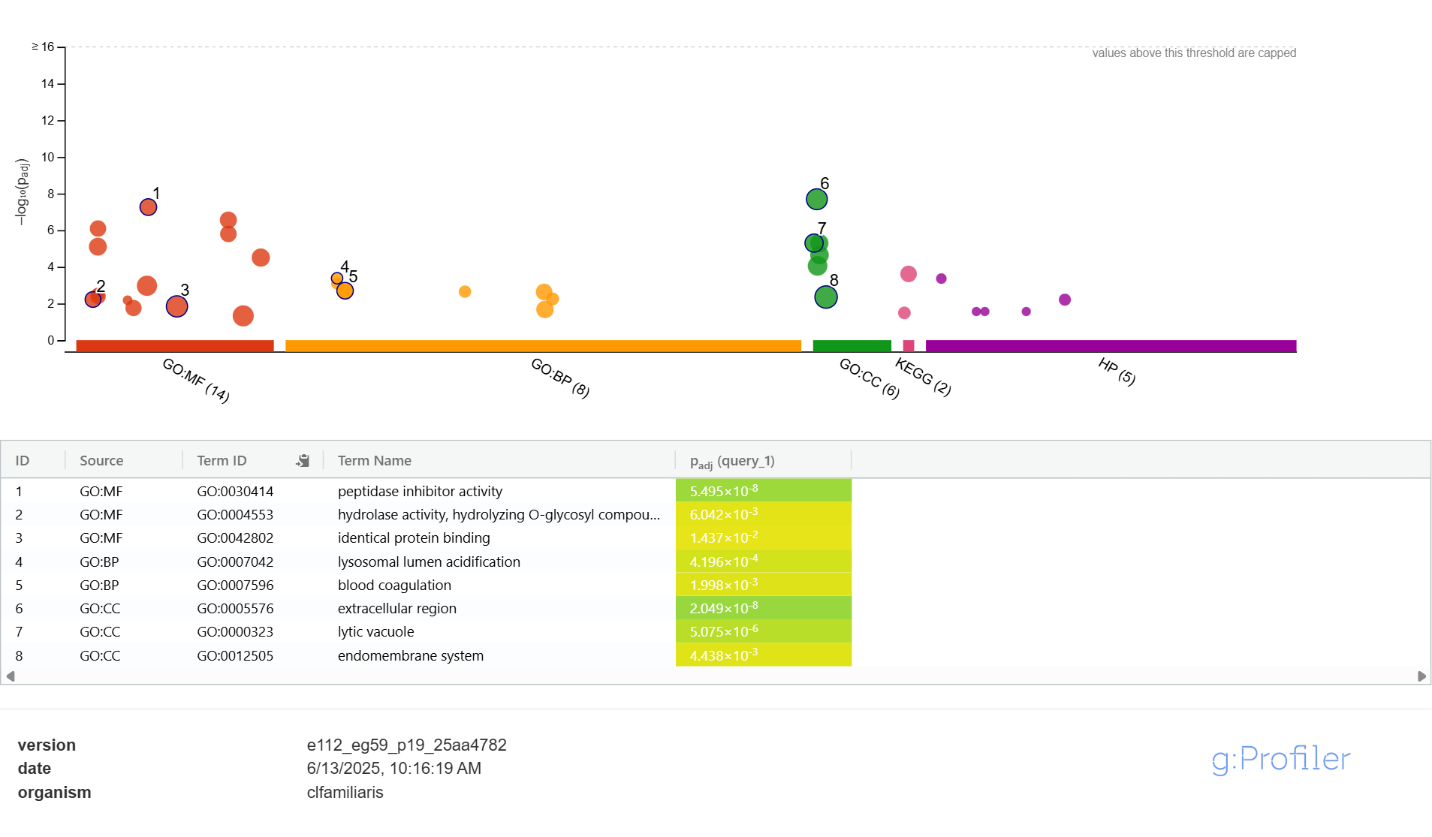


**Supplemental Figure S2. GO analysis of proteins unique Red Wolf sperm from g:Profiler**


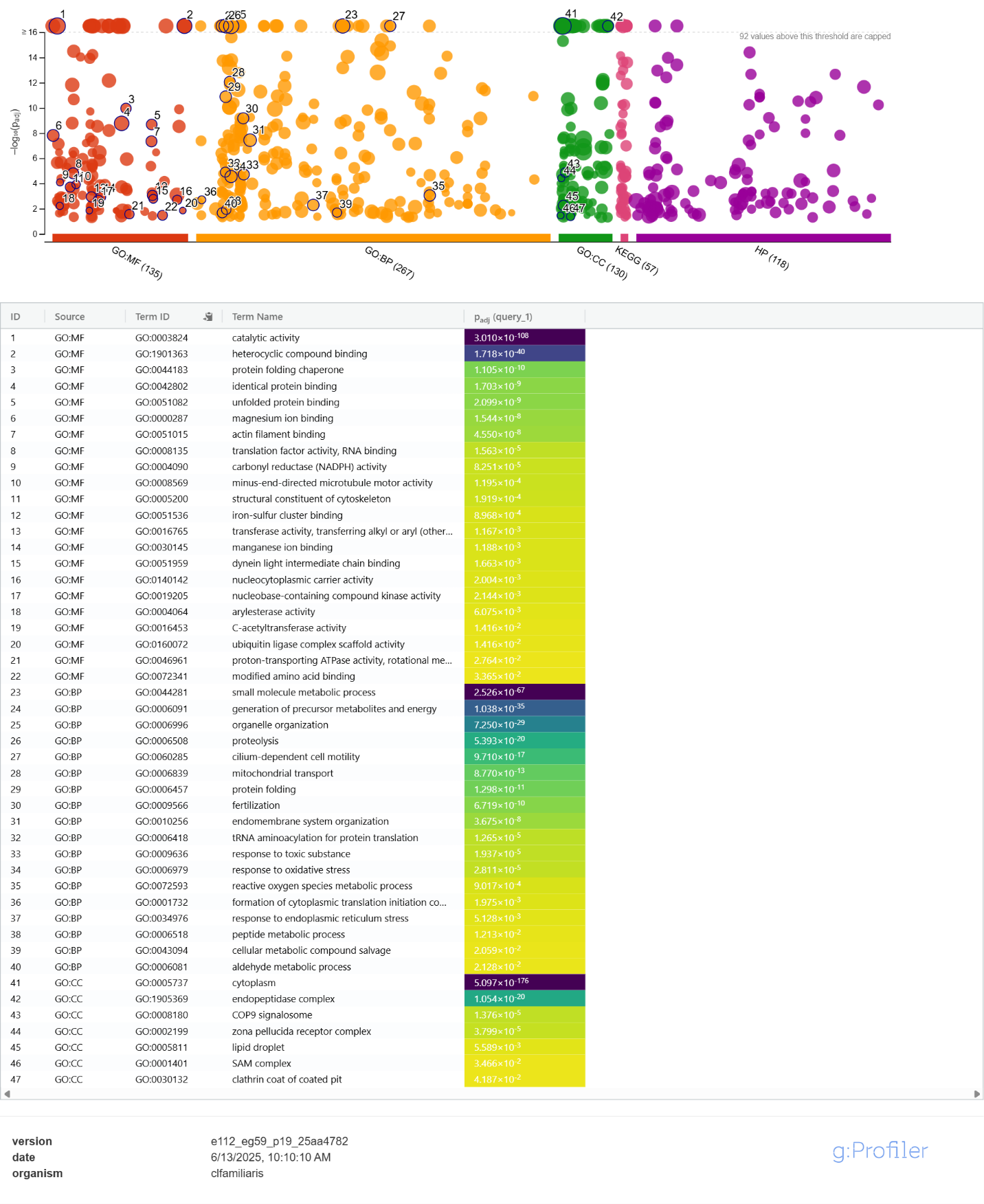


**Supplemental Figure S3. Western blot validation of spermatozoa protein, CRISP2,** with bar graph (signal intensity normalized to Ponceau stain protein amounts per lane) compared with line graph of log2 intensity from mass spectrometry data.
